# Supplementary material for: Systematic transcriptional analysis of human cell lines for gene expression landscape and tumor representation
Source: Nat Commun. 2023 Sep 5;14:5417. doi: 10.1038/s41467-023-41132-w (PMC10480497; doi:10.1038/s41467-023-41132-w)
Supplement: Supplementary file 1 — Supplementary Information [file 41467_2023_41132_MOESM1_ESM.pdf]

Supplementary Materials for

Systematic transcriptional analysis of human cell lines for gene expression landscape and tumor representation

Han Jin[1,†], Cheng Zhang[1,†], Martin Zwahlen[1], Kalle von Feilitzen[1], Max Karlsson[1], Mengnan Shi[1], Meng Yuan[1], Xiya Song[1], Xiangyu Li[1], Hong Yang[1], Hasan Turkez[2], Linn Fagerberg[1], Mathias Uhlén[1,3,*], Adil Mardinoglu[1,4,*]

[1]Department of Protein Science, Science for Life Laboratory, KTH-Royal Institute of Technology, Stockholm 17121, Sweden

[2]Department of Medical Biology, Faculty of Medicine, Atatürk University, Erzurum, Turkey

[3]Department of Neuroscience, Karolinska Institute, Stockholm 17177, Sweden

[4]Centre for Host-Microbiome Interactions, Faculty of Dentistry, Oral & Craniofacial Sciences, King's College London, London, United Kingdom

†These authors contributed equally

*Corresponding authors:

Adil Mardinoglu (adilm@scilifelab.se)

Mathias Uhlén (mathias.uhlen@scilifelab.se)

This PDF file includes:

Supplementary Figures 1 to 7

Other Supplementary Materials include the following:

Supplementary Data 1 to 9

Supplementary Figures

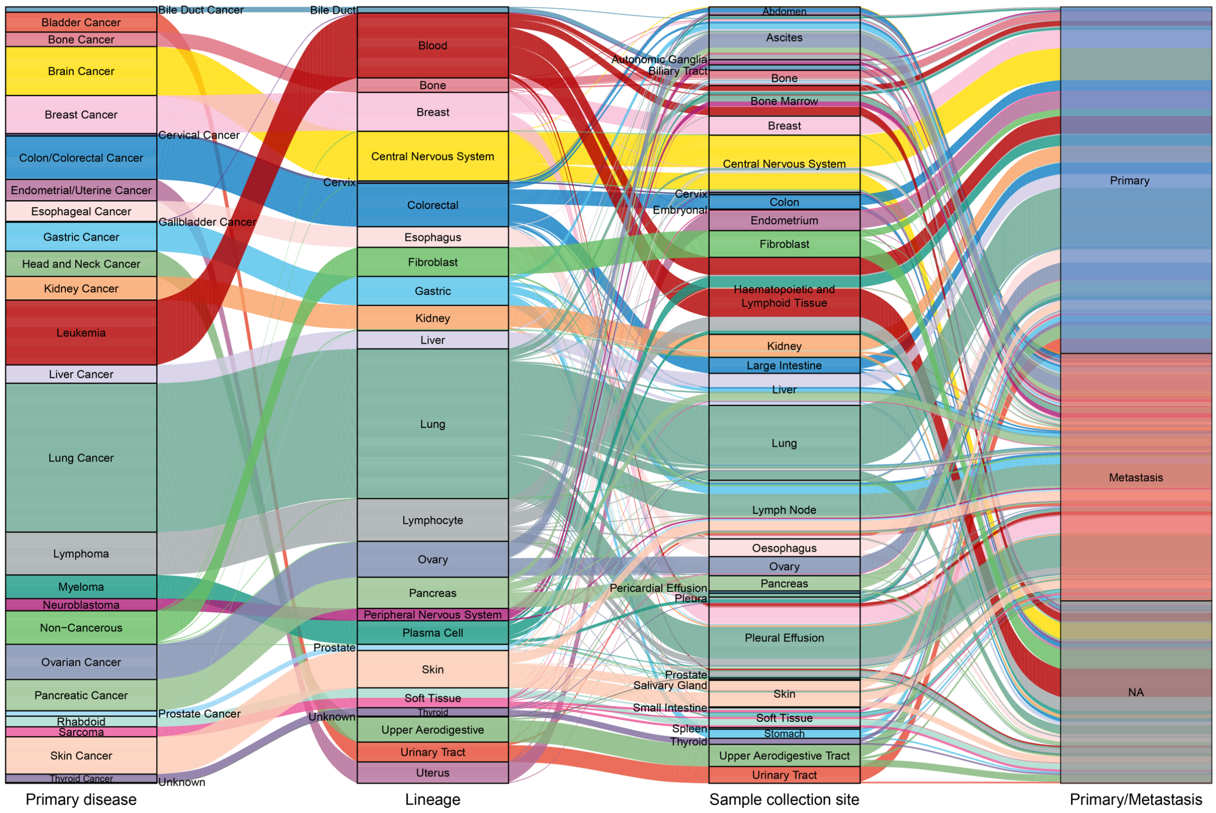

Supplementary Fig. 1. CCLE cell line categorization. Alluvial diagram showing the 1,019 CCLE cell lines categorized by primary disease, lineage, sample collection site, and primary or metastasis, based on the DepMap 2022Q2 annotation. Source data are provided as a Source Data file.

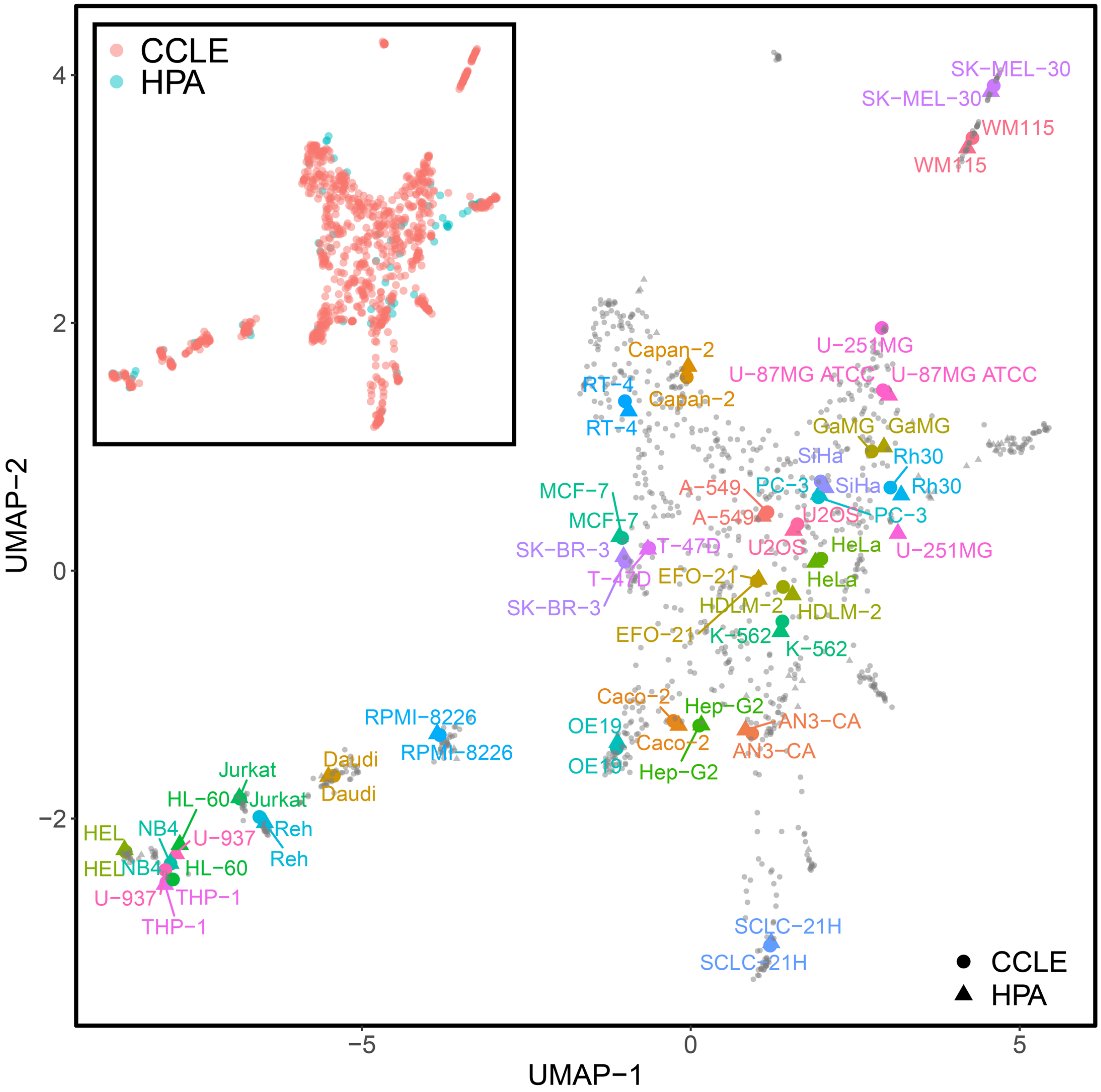

Supplementary Fig. 2. Sample visualization between CCLE and HPA cell lines. UMAP plot showing the relationship between the 1,019 CCLE cell lines and 69 HPA cell lines. Thirty-three pairs of common cell lines between CCLE and HPA are shown in different colors and are textually annotated. For the upper left figure, CCLE and HPA cell lines are denoted in different colors. Source data are provided as a Source Data file.

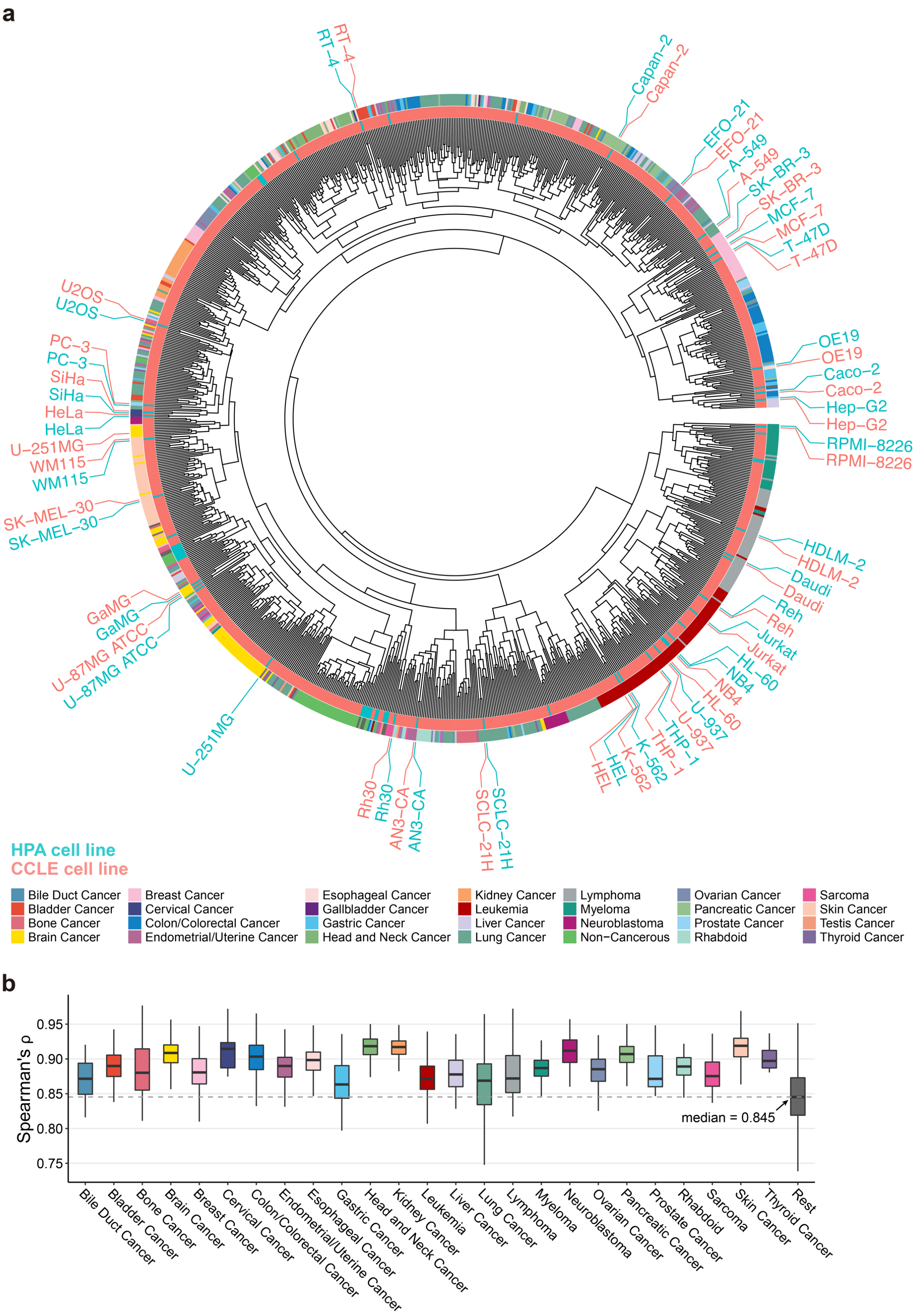
a
RT−4
Capan−2
EFO−21
A−549
SK−BR−3
MCF−7
T−47D
U2OS
PC−3
SiHa
HeLa
U−251MG
WM115
SK−MEL−30
OE19
Caco−2
Hep−G2
RPMI−8226
HDLM−2
Daudi
Reh
Jurkat
HL−60
NB4
U−937
THP−1
K−562
HEL
GaMG
U−87MG ATCC
Rh30
AN3−CA
SCLC−21H
HPA cell line
CCLE cell line
Bile Duct Cancer
Bladder Cancer
Bone Cancer
Brain Cancer
Breast Cancer
Cervical Cancer
Colon/Colorectal Cancer
Endometrial/Uterine Cancer
Esophageal Cancer
Gallbladder Cancer
Gastric Cancer
Head and Neck Cancer
Kidney Cancer
Leukemia
Liver Cancer
Lung Cancer
Lymphoma
Myeloma
Neuroblastoma
Non−Cancerous
Ovarian Cancer
Pancreatic Cancer
Prostate Cancer
Rhabdoid
Sarcoma
Skin Cancer
Testis Cancer
Thyroid Cancer
b
Spearman's ρ
0.95
0.90
0.85
0.80
0.75
median = 0.845
Bile Duct Cancer
Bladder Cancer
Bone Cancer
Brain Cancer
Breast Cancer
Cervical Cancer
Colon/Colorectal Cancer
Endometrial/Uterine Cancer
Esophageal Cancer
Gastric Cancer
Head and Neck Cancer
Kidney Cancer
Leukemia
Liver Cancer
Lung Cancer
Lymphoma
Myeloma
Neuroblastoma
Ovarian Cancer
Pancreatic Cancer
Prostate Cancer
Rhabdoid
Sarcoma
Skin Cancer
Thyroid Cancer
Rest

Supplementary Fig. 3. Sample correlation between CCLE and HPA cell lines. a Circular dendrogram showing the correlation between 1,019 CCLE cell lines and 69 HPA cell lines. Cell lines were hierarchically clustered based on the distance converted from Spearman's correlation coefficient between cell line transcriptomics (1 – Spearman's ρ), with the agglomeration method being set as "complete-linkage". **b** Correlations between cell lines from the same disease compared to the cell lines from different diseases ("Rest"). Statistical significance was evaluated by one-sided Wilcoxon rank-sum test to investigate if the correlations between cell lines from the same disease were significantly higher than the cell lines from different diseases ("Rest"). The number of correlations (n) and p-value (p): Bile Duct Cancer (n = 21; p = 2.39E-04), Bladder Cancer (n = 351; p = 7.66E-118), Bone Cancer (n = 190; p = 1.99E-34), Brain Cancer (n = 2,278; p = 0), Breast Cancer (n = 1,378; p = 2.91E-280), Cervical Cancer (n = 10; p = 1.02E-06), Colon/Colorectal Cancer (n = 1,653; p = 0), Endometrial/Uterine Cancer (n = 406; p = 1.37E-118), Esophageal Cancer (n = 378; p = 8.64E-154), Gastric Cancer (n = 703; p = 3.87E-54), Head and Neck Cancer (n = 528; p = 0), Kidney Cancer (n = 465; p = 1.51E-258), Leukemia (n = 4,560; p = 0), Liver Cancer (n = 300; p = 2.44E-59), Lung Cancer (n = 19,503; p = 0), Lymphoma (n = 1,711; p = 3.20E-265), Myeloma (n = 595; p = 7.70E-186), Neuroblastoma (n = 136; p = 7.52E-72), Ovarian Cancer (n = 1,081; p = 1.48E-278), Pancreatic Cancer (n = 861; p = 0), Prostate Cancer (n = 28; p = 3.60E-07), Rhabdoid (n = 91; p = 3.66E-32), Sarcoma (n = 105; p = 2.93E-22), Skin Cancer (n = 1,326; p = 0), Thyroid Cancer (n = 55; p = 4.06E-14), Rest (n = 478,940). The lower, middle, and upper hinges correspond to the 25th, 50th, and 75th percentiles. The upper whisker extends from the hinge to the largest value no further than 1.5 * IQR from the hinge (where IQR is the inter-quartile range, or distance between the first and third quartiles). The lower whisker extends from the hinge to the smallest value at most 1.5 * IQR of the hinge. Source data are provided as a Source Data file.

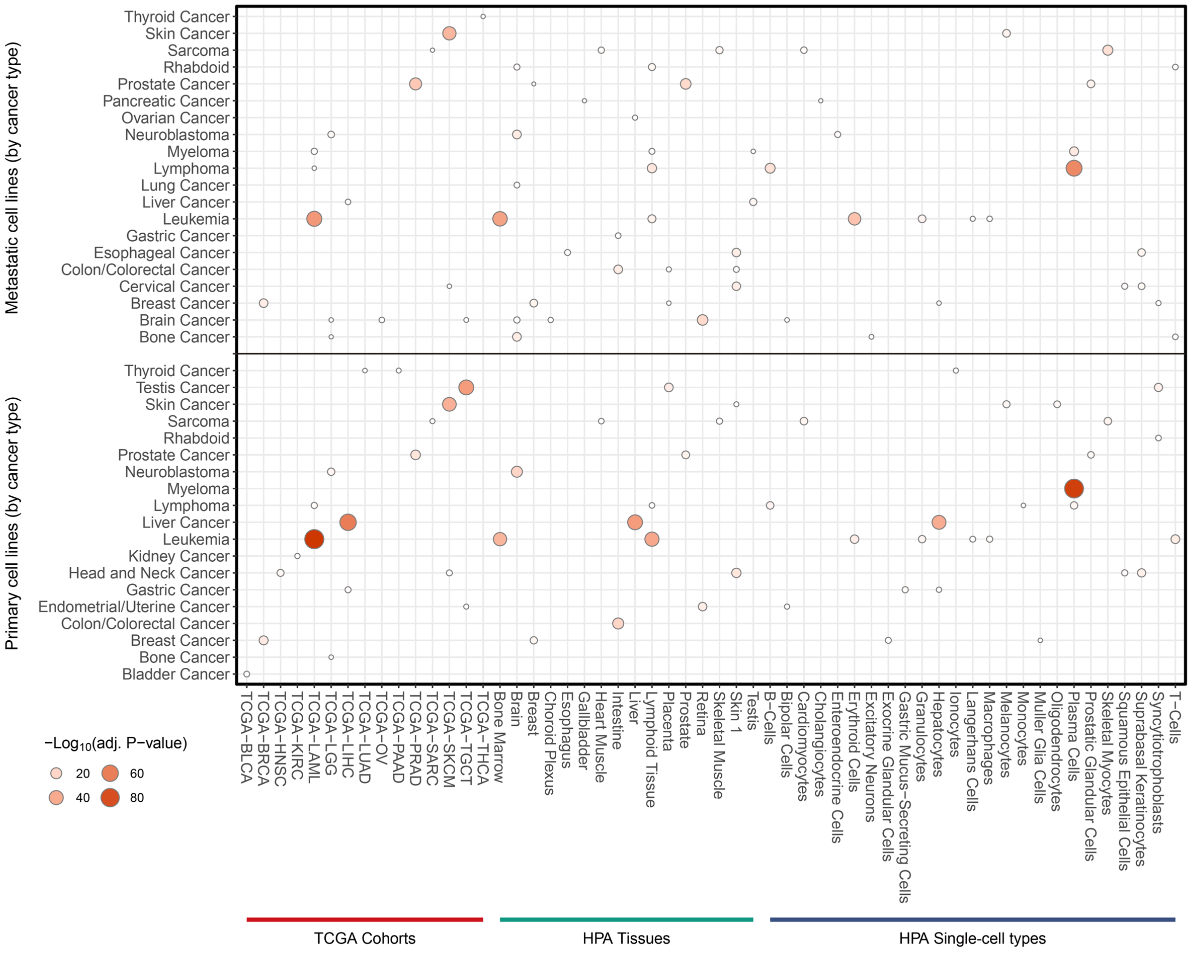

Supplementary Fig. 4. Comparison of gene expression in human cancer cell lines with TCGA cohorts, HPA tissues, and single-cell types. Dot plots showing the significance (evaluated by hypergeometric testing) of the overlap between the enriched genes in TCGA cohorts, HPA tissues, and single-cell types (x-axis) and in CLDs consisting of only primary cell lines (y-axis, lower part) or metastatic cell lines (y-axis, upper part). For both primary and metastatic cell line analyses, non-significant overlaps (adj. P-value > 0.05) are not shown in the figures, and CLDs that are not significantly overlapped with any TCGA cohorts, tissues, and single-cell types, or the other way around, are removed. Source data are provided as a Source Data file.

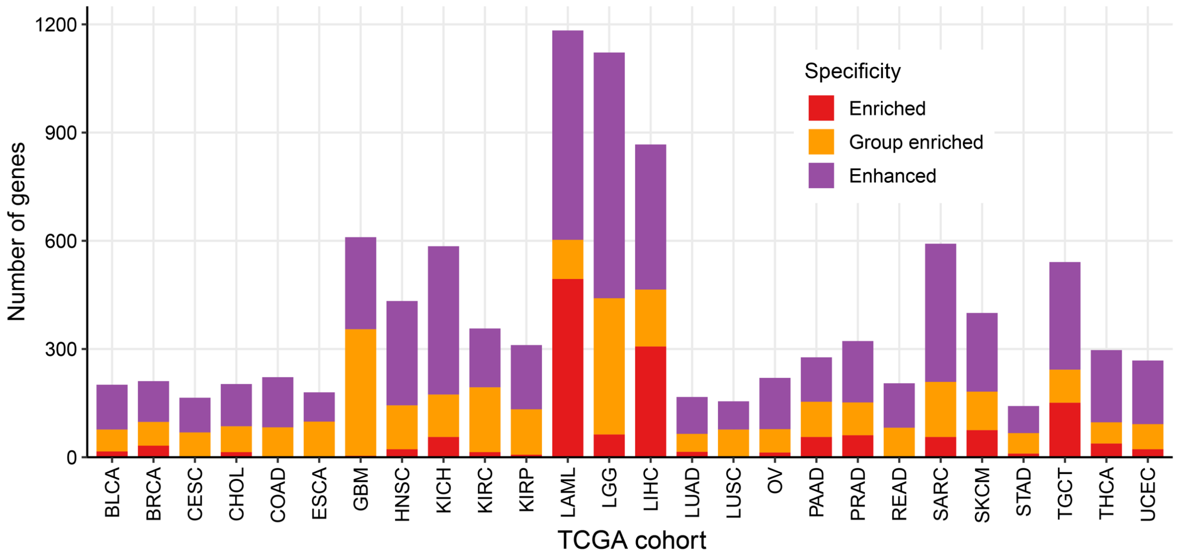

Supplementary Fig. 5. Gene signature of the TCGA cancer cohorts. Bar plot showing the number of elevated genes in each TCGA cohort. Source data are provided as a Source Data file.

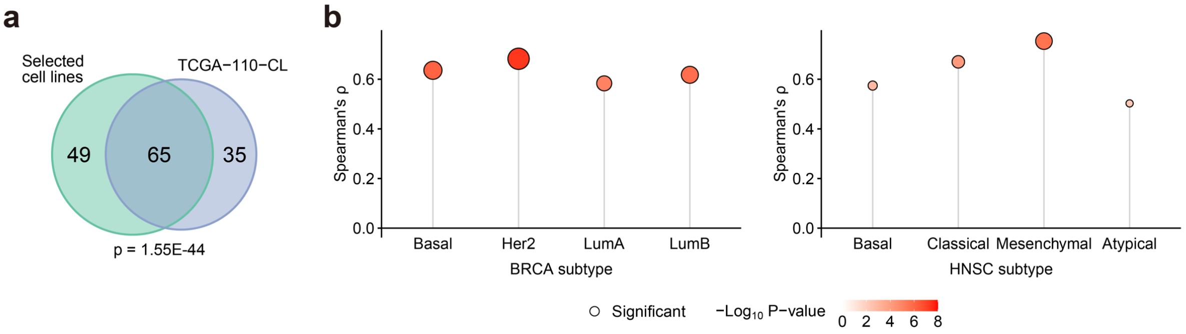

Supplementary Fig. 6. Comparison of selected cell lines with TCGA-110-CL. a Venn diagram showing the overlap between cell lines selected in this study and in the TCGA-110-CL. Statistical significance was evaluated by hypergeometric testing. **b** Spearman's correlation (with significance evaluated in R cor.test() function by default) between the rank of the cell lines in this study and in the TCGA-110-CL study for the TCGA subtypes BRCA and HNSC. Source data are provided as a Source Data file.

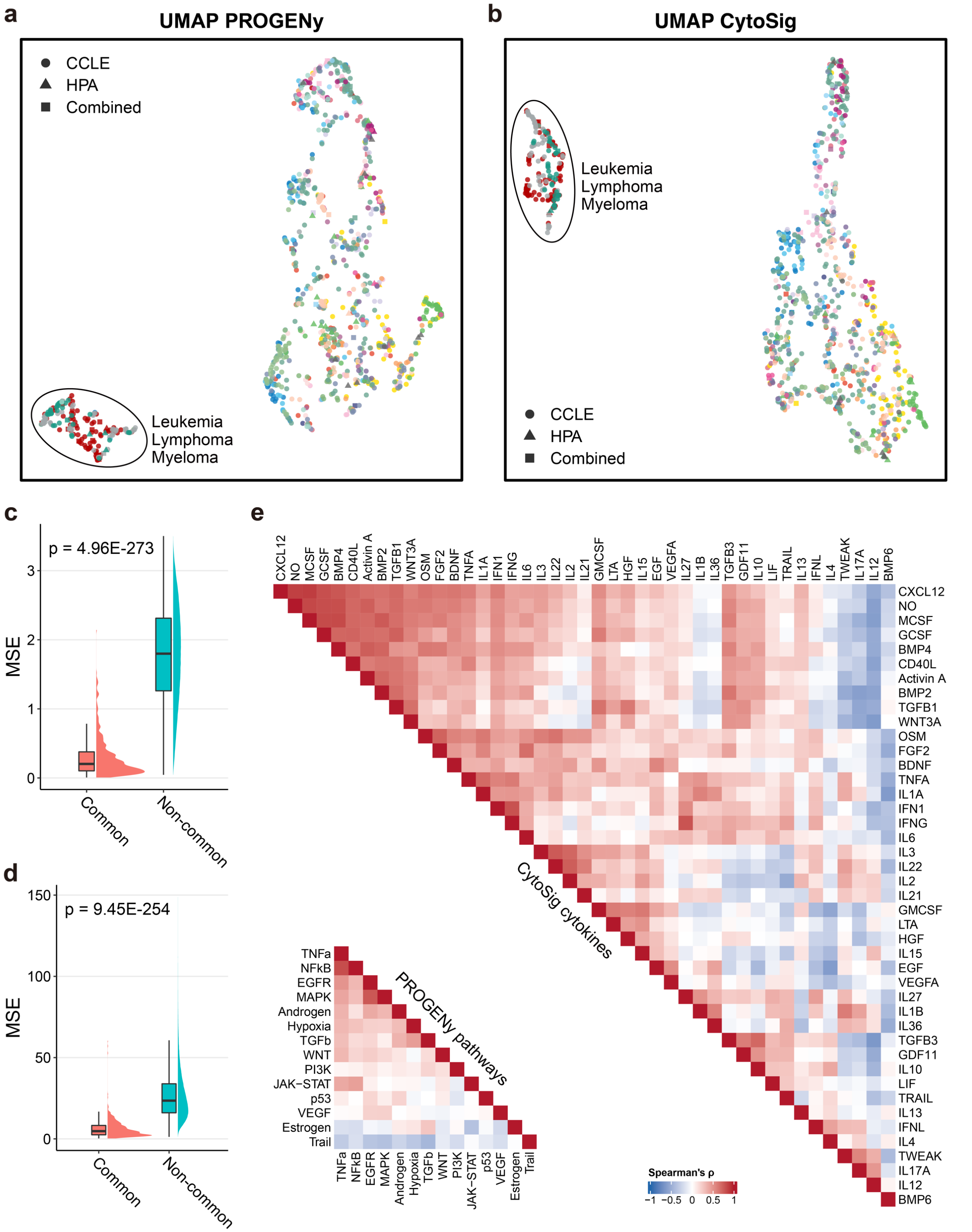

Supplementary Fig. 7. Cancer-related pathway and cytokine activity in human cell lines. a-b UMAP plots showing the relationship between the 1,055 unique cell lines based on the PROGENy pathway activity (**a**) and CytoSig cytokine activity (**b**). **c-d** Boxplots showing the mean square errors (MSE) of common and non-common cell lines between HPA+CCLE and Genentech

datasets (n = 461 MSE values from common cell lines; n = 212,060 MSE values from non-common cell lines) based on the pathway (**c**) and cytokine activity (**d**). Statistical significance was evaluated by two-sided Wilcoxon rank-sum test. The lower, middle, and upper hinges correspond to the 25th, 50th, and 75th percentiles. The upper whisker extends from the hinge to the largest value no further than 1.5 * IQR from the hinge (where IQR is the inter-quartile range, or distance between the first and third quartiles). The lower whisker extends from the hinge to the smallest value at most 1.5 * IQR of the hinge. Significance was evaluated by two-sided Wilcoxon rank-sum test. **e** Correlations between the 14 PROGENy pathways (lower left) and between the 43 CytoSig cytokines (upper right) based on the 1,055 analyzed cell lines. Source data are provided as a Source Data file.
