# Supplementary material for: Systematic transcriptional analysis of human cell lines for gene expression landscape and tumor representation
Source: Nat Commun. 2023 Sep 5;14:5417. doi: 10.1038/s41467-023-41132-w (PMC10480497; doi:10.1038/s41467-023-41132-w)
Supplement: Supplementary file 11 — Reporting Summary [file 41467_2023_41132_MOESM11_ESM.pdf]

nature portfolio

Corresponding author(s): Adil Mardinoglu
Mathias Uhlen

Last updated by author(s): Aug 22, 2023

Reporting Summary

Nature Portfolio wishes to improve the reproducibility of the work that we publish. This form provides structure for consistency and transparency in reporting. For further information on Nature Portfolio policies, see our Editorial Policies and the Editorial Policy Checklist.

Statistics

For all statistical analyses, confirm that the following items are present in the figure legend, table legend, main text, or Methods section.

n/a	Confirmed	
☐	☒	The exact sample size (*n*) for each experimental group/condition, given as a discrete number and unit of measurement
☐	☒	A statement on whether measurements were taken from distinct samples or whether the same sample was measured repeatedly
☐	☒	The statistical test(s) used AND whether they are one- or two-sided<br>*Only common tests should be described solely by name; describe more complex techniques in the Methods section.*
☒	☐	A description of all covariates tested
☐	☒	A description of any assumptions or corrections, such as tests of normality and adjustment for multiple comparisons
☐	☒	A full description of the statistical parameters including central tendency (e.g. means) or other basic estimates (e.g. regression coefficient) AND variation (e.g. standard deviation) or associated estimates of uncertainty (e.g. confidence intervals)
☐	☒	For null hypothesis testing, the test statistic (e.g. *F*, *t*, *r*) with confidence intervals, effect sizes, degrees of freedom and *P* value noted<br>*Give P values as exact values whenever suitable.*
☒	☐	For Bayesian analysis, information on the choice of priors and Markov chain Monte Carlo settings
☒	☐	For hierarchical and complex designs, identification of the appropriate level for tests and full reporting of outcomes
☒	☐	Estimates of effect sizes (e.g. Cohen's *d*, Pearson's *r*), indicating how they were calculated

Our web collection on statistics for biologists contains articles on many of the points above.

Software and code

Policy information about availability of computer code

Data collection	SRA Toolkit (v2.11.3) was used to download the CCLE RNA-seq data from the Sequence Read Archive (SRA). Genentech RNA-seq data were download using pyEGA3 (v4.0.3). TCGA data were downloaded by the R package TCGAbiolinks (v2.27.2).
Data analysis	RNA-seq fastq files were processed using Kallisto (v0.46.2). Gene expression was assembled using the R package tximport (v1.22.0). Gene expression was normalized by the R package NOISeq (v2.38.0) and DESeq2 (v1.34.0). Machine learning analysis was performed using the scikit-learn package (v1.0.2). Tumor purity was analyzed using the R package estimate (v1.0.13). Gene set overrepresentation analysis was performed using the R package clusterProfiler (v4.2.2). Gene set enrichment analysis was performed using the R package fgsea (v1.20.0). Pathway analysis was performed using the R package progeny (v1.16.0) and decoupleR (v2.0.1). Cytokine analysis was performed using the package CytoSig (v0.0.2). PCA was performed using the R package pcaMethods (v1.86.0). UMAP was performed using the R package swot (v0.1.11). R (v4.1.2) was used for data analysis. Custom code used in this study can be found here: https://doi.org/10.5281/zenodo.8221434.

For manuscripts utilizing custom algorithms or software that are central to the research but not yet described in published literature, software must be made available to editors and reviewers. We strongly encourage code deposition in a community repository (e.g. GitHub). See the Nature Portfolio guidelines for submitting code & software for further information.

Data

Policy information about availability of data

All manuscripts must include a data availability statement. This statement should provide the following information, where applicable:

- Accession codes, unique identifiers, or web links for publicly available datasets
- A description of any restrictions on data availability
- For clinical datasets or third party data, please ensure that the statement adheres to our policy

The CCLE publicly available RNA-seq data used in this study are available in the Sequence Read Archive (SRA) database under accession code PRJNA523380 [https://www.ncbi.nlm.nih.gov/bioproject/?term=PRJNA523380][1]. The RNA-seq data of the HPA cell lines generated for this study have been deposited in the Gene Expression Omnibus database under accession code GSE240542 [https://www.ncbi.nlm.nih.gov/geo/query/acc.cgi?acc=GSE240542]. The Genentech RNA-seq data is available under restricted access, access can be obtained by request from the European Genome-phenome Archive (EGA) under accession number EGAS00001000610 [https://ega-archive.org/studies/EGAS00001000610][2]. The TCGA data are publicly available at https://portal.gdc.cancer.gov/. The processed gene expression data for CCLE and HPA cell lines are available to download on the Human Protein Atlas resource download page [https://v22.proteinatlas.org/about/download]. The remaining data are available within the Article, Supplementary Information or Source Data file.

1 Ghandi, M. et al. Next-generation characterization of the Cancer Cell Line Encyclopedia. Nature 569, 503-508, doi:10.1038/s41586-019-1186-3 (2019).
2 Klijn, C. et al. A comprehensive transcriptional portrait of human cancer cell lines. Nature Biotechnology 33, 306-312, doi:10.1038/nbt.3080 (2015).

Human research participants

Policy information about studies involving human research participants and Sex and Gender in Research.

Reporting on sex and gender	N.A.
Population characteristics	N.A.
Recruitment	N.A.
Ethics oversight	N.A.

Note that full information on the approval of the study protocol must also be provided in the manuscript.

Field-specific reporting

Please select the one below that is the best fit for your research. If you are not sure, read the appropriate sections before making your selection.

[x] Life sciences [ ] Behavioural & social sciences [ ] Ecological, evolutionary & environmental sciences

For a reference copy of the document with all sections, see nature.com/documents/nr-reporting-summary-flat.pdf

Life sciences study design

All studies must disclose on these points even when the disclosure is negative.

Sample size	In this study, a total of 69 cell lines from HPA and 1,019 cell lines from CCLE were analyzed by RNA-seq. Cell line RNA-seq dataset from Genentech (n = 610) was used for the validation of pathway and cytokine scores. In Figure 3d, statistical analysis was made to estimate the differences between the correlations between primary cancer cell lines (n = 322) and the correlations between metastatic cancer cell lines (n = 447). Each group in Figure 3d has around 7,000 data points. The same holds for Supplementary Figures 3b (on average 19,909 data points in each group) and 7c-d (n = 461 and 21,060 data points in the two groups, respectively). Given that sufficient cell line samples and data points were used in these analyses, the reliability of the statistical analysis is assured.
Data exclusions	No data were excluded from the analysis.
Replication	The results can be reproduced by running the scripts.
Randomization	This study is not relevant to clinical trials and animal experiments. All the RNA-seq data are derived from human cell lines and are publicly available. No randomization is performed in this study.
Blinding	This study is not relevant to clinical trials and animal experiments. All the RNA-seq data are derived from human cell lines and are publicly available. No blinding is performed in this study.

Reporting for specific materials, systems and methods

We require information from authors about some types of materials, experimental systems and methods used in many studies. Here, indicate whether each material, system or method listed is relevant to your study. If you are not sure if a list item applies to your research, read the appropriate section before selecting a response.

Materials & experimental systems

n/a	Involved in the study
☒	☐ Antibodies
☒	☐ Eukaryotic cell lines
☒	☐ Palaeontology and archaeology
☒	☐ Animals and other organisms
☒	☐ Clinical data
☒	☐ Dual use research of concern

Methods

n/a	Involved in the study
☒	☐ ChIP-seq
☒	☐ Flow cytometry
☒	☐ MRI-based neuroimaging
