# Supplementary material for: Systematic transcriptional analysis of human cell lines for gene expression landscape and tumor representation
Source: Nat Commun. 2023 Sep 5;14:5417. doi: 10.1038/s41467-023-41132-w (PMC10480497; doi:10.1038/s41467-023-41132-w)
Supplement: Supplementary file 12 — Description of Additional Supplementary Files [file 41467_2023_41132_MOESM12_ESM.pdf]

Title: Supplementary Data 1.
Description: Annotation of 69 HPA cell lines.

Title: Supplementary Data 2.
Description: Annotation of 1,019 CCLE cell lines.

Title: Supplementary Data 3.
Description: Gene set overrepresentation analysis of the essential genes based on Gene Ontology.

Title: Supplementary Data 4.
Description: Mapping table between TCGA cohorts and diseases of analyzed cancer cell lines.

Title: Supplementary Data 5.
Description: Cell line prioritization for TCGA cohorts.

Title: Supplementary Data 6.
Description: Cell line prioritization for TCGA cohorts at the pathologic stage level.

Title: Supplementary Data 7.
Description: Cell line prioritization for TCGA cohorts at the molecular subtype level.

Title: Supplementary Data 8.
Description: Results of PROGENy pathway and CytoSig cytokine analysis.

Title: Supplementary Data 9.
Description: Cell line prioritization for TCGA cohorts based on PROGENy and CytoSig activity.
